# *AHRR* (cg05575921) methylation extent of leukocyte DNA and lung cancer survival

**DOI:** 10.1371/journal.pone.0211745

**Published:** 2019-02-07

**Authors:** Katja Kemp Jacobsen, Jakob Sidenius Johansen, Anders Mellemgaard, Stig Egil Bojesen

**Affiliations:** 1 Department of Technology, Faculty of Health and Technology, University College Copenhagen, Copenhagen, Denmark; 2 Department of Oncology, Herlev and Gentofte Hospital, Copenhagen University Hospital, Copenhagen, Denmark; 3 Department of Clinical Biochemistry, Herlev and Gentofte Hospital, Copenhagen University Hospital, and Faculty of Health and Medical Sciences, University of Copenhagen, Copenhagen, Denmark; University of South Alabama Mitchell Cancer Institute, UNITED STATES

## Abstract

**Background:**

Prior studies have shown that *AHRR* (cg05575921) hypomethylation may be a marker of smoking, lung cancer risk and potentially lung cancer survival (in some lung cancer subtypes). It is unknown if *AHRR* (cg05575921) hypomethylation is associated with reduced survival among lung cancer patients.

**Methods:**

In bisulfite treated leukocyte DNA from 465 lung cancer patients from the Copenhagen prospective lung cancer study, we measured *AHRR* (cg05575921) methylation. 380 died during max follow-up of 4.4 years. Cox proportional hazard models were used to analyze survival as a function of *AHRR* (cg05575921) methylation.

**Results:**

We observed the expected inverse correlation between cumulative smoking and *AHRR* methylation, as methylation (%) decreased (Coefficient -0.03; 95% confidence interval, -0.04- -0.02, p = 8.6x10^-15^) for every pack-year. Cumulative smoking > 60 pack-years was associated with reduced survival (hazard ratio and 95% confidence interval 1.48; 1.05–2.09), however, *AHRR* (cg05575921) methylation was not associated with survival when adjusted for sex, body mass index, smoking status, ethnicity, performance status, TNM Classification, and histology type of lung cancer.

**Conclusion:**

*AHRR* (cg05575921) methylation is linked to smoking but does not provide independent prognostic information in lung cancer patients.

## Introduction

Lung cancer is the leading cause of cancer-related death worldwide [1]. The overall 5-year survival of lung cancer patients remains below 20% [2–3], mainly because tumors are diagnosed at late stages [4]. Patients with advanced stage lung cancer are often treated with systemic chemotherapy or immunotherapy [5] with a limited effect on the prognosis. Introduction of medical therapies targeted to specific oncogenic pathways might have the potential to improve survival for a subset of these patients [6]. However, biomarkers for identification of patients that specifically benefits from therapy are needed.

Alteration in DNA methylation is an epigenetic modification that are common in human malignancies [7]. Tobacco smoke alter DNA methylation and thereby influence disease through complex mechanisms [8–9]. Altered DNA methylation is caused by tobacco smoking with hypomethylation at cg05575921 located at the third intron of the aryl-hydrocarbon receptor repressor (*AHRR*) [10–18]. In the general population, *AHRR* (cg05575921) hypomethylation of leukocyte DNA is associated with both former and current smoking status, daily cigarette consumption, cumulative smoking and smoking duration in both former and current smokers, and time since quitting in former smokers [19]. This suggests that blood based *AHRR* (cg05575921) methylation extent can be used as an objective measure of long-term smoking exposure.

Tobacco smoking is a prognostic marker among lung cancer patients overall [20], and after treatment with EGFR-tyrosine kinase inhibitors [21]. Importantly, *AHRR* (cg05575921) methylation extent has been linked to lung cancer risk [15,17,19], and seems to be a more accurate risk estimator of lung cancer than self-reported smoking [19]. Additionally, DNA methylation plays a role in lung tumor initiation and progression [22–23]. It is unclear if *AHRR* methylation in itself is a cause of increased lung cancer risk. However, hypomethylation of *AHRR* has been associated with higher *AHRR* expression in monocytes [24], lymphoblasts and pulmonary macrophages [25], entailing decreased capability to metabolize and remove harmful agents such as polycyclic aromatic hydrocarbons, that are contained in tobacco smoke, potentially leading to development of tobacco-related lung damage.

We hypothesized that *AHRR* (cg05575921) hypomethylation, as a strong biomarker of long-term smoking behavior, offers additional information beyond that offered by self-reported smoking to identify lung cancer patients that specifically benefits from therapy. Thus, we examined the association between *AHRR* (cg05575921) hypomethylation in bisulfite treated leukocyte DNA and survival among lung cancer patients from the Lung Cancer Study (LUCAS), also referred to as the Copenhagen study [26].

## Materials and methods

### Study cohort

465 consecutive individuals of the Copenhagen Study [26] who had lung cancer diagnosed at Department of Respiratory Medicine, Bispebjerg University Hospital in Denmark between February 2012 and December 2013. Individuals were excluded if ECOG performance status was above 3 at first assessment by the oncologist. Cancer diagnose was cytologically or histologically verified, and staged according to the TNM Classification of Malignant Tumors (TNM).

At first assessment, individuals were asked about smoking status. Former smoking was defined as no smoking within last 6 months. Smoking status included number of daily consumed cigarettes, cheroots, cigars or pipes, age at smoking initiation and cessation. Pack-years was consumption of 20 cigarettes or equivalent per day for 1 year. Body mass index (BMI) was calculated as measured weight in kilos divided by measured height in meters squared, performance status was assessed according to ECOG classification, and a blood sample were drawn.

The study was approved by Herlev and Gentofte Hospital, the Danish Data Inspection Agency (KF100.2039/91), and was conducted according to the Declaration of Helsinki. Written informed consent was obtained from all participants.

### *AHRR* hypomethylation

The *AHRR* (cg05575921) methylation extent was measured in duplicate samples using a Taqman assay developed in our own laboratory, and validated against results from pyrosequencing. Bisulphite treated leucocyte DNA drawn from peripheral blood, was amplified using forward and reverse PCR primers, which were designed to bind to DNA around the cg05575921 site on sequences without genetic or possible CpG methylation variation. Details of the *AHRR* methylation measurements have been published previously [19].

### Survival

Date of death was found by linkage with the Danish Civil Registration System until January 2017. There was no loss to follow-up. The underlying cause of death until March 2017 were obtained from the Danish Register of Causes of Death.

### Statistical analysis

We used STATA/SE 15.1. All 465 individuals were categorized into ranked quantiles of *AHRR* (cg05575921) methylation extent (%) with the highest methylation quantile defined as the reference category, presumably representing individuals least exposed to smoking. We showed data in quantiles for simple illustrative presentation of the results; however, analyses on *AHRR* (cg05575921) methylation extent (%) categorized into deciles and on a continuous scale are also shown. We used Kruskal Wallis test and Pearsons X^2^-test to test for trend of *AHRR* methylation through all included variables.

Individual follow-up time began at baseline, which was defined as the date of lung cancer diagnosis by biopsy, and ended the day of death or 31 January 2017. Minimum, median and maximum follow-up time were 11 days, 332 days, and 4.4 years for all-cause mortality, giving a total of 688.495 person-years and 380 deaths (81.7%).

Cox proportional hazard regression model using length of follow-up as the time metric estimated hazard ratios (HRs) and 95% confidence intervals (95% CI) for the association between *AHRR* (cg05575921) methylation (%) and survival in four steps with increasing level of adjustment for potential *a priori* selected confounders. 1) a crude model, 2) a model adjusted for age at lung cancer diagnosis and sex 3) a multivariable model additionally adjusted for body mass index (kg/m^2^) (BMI), ethnicity (Europeans/others), Classification of Malignant Tumors (TNM) (Stage I-IIII), histology of lung cancer (small cell lung cancer, adenocarcinoma, squamous-cell carcinoma, other non-small-cell lung carcinoma (NSCLC), performance status (0–3), 4) a smoking plus model additionally adjusted for smoking status (never/former/current smoker) and cumulative smoking (defined as 20 cigarettes/day per year, calculated from smoking intensity (number of cigarettes a day) and smoking duration (years)). Models were applied for the whole cohort as well as stratified by cause of death (lung cancer, cardiac death, respiratory death or other cancer) and treatment. We performed test for trend by using the continuous variable in the Cox model. We checked for the proportional hazards assumption for all variables based on scaled Schoenfeld residuals. Potential interactions between *AHRR* (cg05575921) methylation (per 1%) and survival were tested for by introducing interaction terms into the Cox model, and tested by the Walds test. Survival curves were generated using Kaplan-Meier analysis method, and the log rank test was used to examine differences in survival between the ranked quantiles of *AHRR* (cg05575921) methylation.

In a sensitivity analysis, we included 461 individuals without lung cancer from the 1991–1994 examination of the Copenhagen City Heart Study [19], a prospective cohort study of the Danish general population (S1 Table). Individuals were selected after 1:1 matching with the lung cancer patients on body mass index, age, cumulative smoking and sex. Individual follow-up time for all-cause mortality began at the date of examination, and ended the day of death or 14 November 2014. We did this to assess potential interaction between *AHRR* (cg05575921) methylation and lung cancer status on survival. Potential interactions were tested for by introducing interaction terms into the Cox model, and tested by the Walds test.

To validate the methylation measurement, we used linear regression analysis to measure expected association between cumulative smoking and *AHRR* (cg05575921) methylation. To assess the expected association between all-cause mortality and cumulative smoking, treatment, recurrence, ECOG performance status and TNM-status, we used the Cox model.

## Results

*A priori* we calculated the power of the study, and the study has 80% power to detect reduced survival of at least 6% every time *AHRR* (cg05575921) hypomethylation is reduced by 1% (SD = 2.17%).

The study population consisted of 465 lung cancer cases (Table 1), slightly more women (51.8%) than men with a median age of 68.3 years. Median of *AHRR* (cg05575921) methylation was 56.0%. A majority of cases was European (97.4%), had body mass index between 18.5 and 24.9 (kg/m^2^), were former smokers (56.5%), TNM stage IIII (52.0%), at performance status 1 (40.0%), had adenocarcinoma (54.4%), received platinium-based chemotherapy as first oncological treatment (65.2%), had one line of treatment (65.0%) and had no recurrence of their lung cancer within the follow-up period (79.1%).

**Table 1:** Baseline characteristics of 465 patients with lung cancer by leukocyte DNA methylation of *AHRR* (cg05575921), quantiles.

	Total	Leukocyte DNA methylation of *AHRR* (cg05575921), quantiles				
		1^st^ (highest) 57.4–63.4%	2^nd^ 56.0–57.4%	3^rd^ 54.9–56.0%	4^th^ (lowest) 50.0–54.9%	p-value^a^
**Total (%)**	465	123 (26.5)	109 (23.4)	120 (25.8)	113 (24.3)	-
**Age years**	68.3 (62.7–74.7)	70.2 (64.9–77.0)	67.7 (63.2–74.7)	67.9 (61.3–73.0)	66.9 (59.7–73.7)	0.05
***AHRR* (cg05575921) methylation extent (%)**	56.0 (54.9–57.4)	58.6 (58.0–60.0)	56.6 (56.4–56.9)	55.5 (55.1–55.8)	54.0 (53.3–54.3)	NA
**Sex**						
Men	224 (48.2)	52 (42.3)	57 (52.3)	62 (51.7)	53 (46.9)	
Women	241 (51.8)	71 (57.7)	52 (47.7)	58 (48.3)	60 (53.1)	0.37
**Ethnicity,**						
European	453 (97.4)	118 (95.9)	107 (98.2)	116 (96.7)	112(93.8)	
Others	12 (2.6)	5 (4.1)	2 (1.8)	4 (3.3)	1 (0.9)	0.41
**Body mass index (kg/m**^**2**^**)**						
<18.5	50 (10.8)	2 (1.6)	12 (11.0)	18 (15.0)	18 (15.9)	
18.5–24.9	223 (48.0)	62 (50.4)	49 (45.0)	53 (44.2)	59 (52.2)	
25.0–29.9	111 (23.9)	31 (25.2)	29 (26.6)	32 (26.7)	19 (16.8)	
≥30.0	37 (8.0)	16 (13.0)	7 (6.4)	8 (6.7)	6 (5.3)	
Missing, n (%)	44 (9.4)	12 (9.8)	12 (11.0)	9 (7.5)	11 (9.7)	0.004
**Smoking status**						
Never	28 (6.0)	24 (19.5)	1 (0.9)	2 (1.7)	1 (0.9)	
Former (>6 months ago)	262 (56.5)	87 (70.7)	79 (72.5)	56 (46.7)	40 (35.4)	
Current	174 (37.5)	11 (8.9)	29 (26.6)	62 (51.6)	72 (64.6)	
Missing, n (%)	1 (0.0)	1 (0.8)	0 (0.0)	0 (0.0)	0 (0.0)	2.4x10^-25^
**Cumulative smoking**^**b**^**, pack-years**	40.0 (24. 0–51.4)	22.7 (2.5–40)	41.0 (28–54.2)	43.4 (29.5–53.0)	44.5 (32.4–56.3)	0.0001
Missing	10 (2.2)	5 (4.1)	2 (1.8)	2 (1.7)	1 (0.9)	
**Smoking duration, years**	47.0 (40.0–54.0)	41.5 (27.5–51.2)	48 (42.0–54.0)	49.0 (41.0–56.0)	49.0 (43.3–55.0)	0.0002
Missing, n (%)	100 (21.5)	14 (11.4)	29 (26.7)	31 (25.8)	26 (23.0)	
**Time since smoking cessation, years**	3.3 (0.8–12.4)	10.2 (3.4–18.4)	2.0 (0.6–8.9)	1.0(0.7–4.1)	1.0(0.7–6.1)	0.0001
Missing	35 (7.5)	10 (8.1)	15 (13.7)	8 (6.7)	4 (3.5)	
**TNM Classification of Malignant Tumours (TNM)**						
Stage Ia-b	26 (5.6)	3 (2.5)	8 (7.3)	6 (5.0)	9 (8.0)	
Stage IIa-b	33 (7.1)	6 (4.9)	11 (10.1)	8 (6.7)	8 (7.1)	
Stage IIIa-b	163 (35.1)	35 (28.7)	44 (40.4)	47 (39.2)	40 (35.4)	
Stage IIII	242 (52.0)	78 (63.9)	49 (45.0)	59 (49.2)	56 (49.6)	
Missing, n (%)	1 (0.2)	1 (0.2)	0 (0.0)	0 (0.0)	0 (0.0)	0.16
**Performance status**						
0	158 (34.0)	45 (36.6)	44 (40.4)	35 (29.2)	34 (30.1)	
1	186 (40.0)	50 (40.7)	43 (39.4)	51 (42.5)	42 (37.2)	
2	81 (17.4)	16 (13.0)	16 (14.7)	27 (22.5)	22 (19.5)	
3	35 (7.5)	10 (8.1)	6 (5.5)	6 (5.0)	13 (11.5)	
Missing, n (%)	5 (1.1)	2 (1.6)	0 (0.0)	1 (0.8)	2 (1.8)	0.26
**Histology**						
Small cell lung cancer	78 (16.8)	11 (8.9)	15 (13.8)	25 (20.8)	27 (23.9)	
NSCLC, adenocarcinoma	253 (54.4)	76 (61.8)	58 (53.2)	63 (52.5)	56 (49.6)	
NSCLC, squamous carcinoma	102 (21.9)	25 (20.3)	29 (26.6)	23 (19.2)	25 (22.1)	
NSCLC, other	32 (6.9)	11 (8.9)	7 (6.4)	9 (7.5)	5 (4.4)	0.09
**First treatment for lung cancer**^**c**^						
Platinum-based therapy	303 (65.2)	83 (67.5)	83 (76.2)	81 (67.5)	56 (49.6)	
Therapy,combination^d^	1 (0.2)	0 (0.0)	0 (0.0)	0 (0.0)	1 (0.9)	
Therapy,monotherapy^d^	3 (0.7)	2 (0.0)	1 (0.9)	0 (0.0)	0 (0.0)	
Targeted therapy^e^	3 (0.7)	2 (1.6)	0 (0.0)	0 (0.0)	1 (0.9)	
Immunotherapy	4 (0.9)	2 (1.6)	0 (0.0)	1 (0.8)	1 (0.9)	
No oncological treatment^f^	151 (32.5)	34 (27.6)	25 (22.9)	38 (31.7)	54 (47.8)	0.01
**Recurrence of lung cancer**^**g**^						
No	368 (79.1)	100 (81.3)	81 (74.3)	92 (76.7)	95 (84.1)	
Yes	97 (20.9)	23 (18.7)	28 (25.7)	28 (23.3)	18 (15.9)	0.26
<3 months of treatment start	11 (11.3)	3 (13.0)	4 (14.3)	2 (7.1)	2 (11.1)	
>3 months of treatment start	86 (88.7)	20 (87.0)	24 (85.7)	26 (92.9)	16 (88.9)	0.85
**Total lines of treatment**						
1	204 (65.0)	62 (69.7)	50 (59.5)	53 (64.6)	39 (66.1)	
2	75 (23.9)	14 (15.7)	26 (31.0)	19 (23.2)	16 (27.1)	
3	27 (8.6)	9 (10.1)	7 (8.3)	9 (11.0)	2 (3.4)	
4	5 (1.6)	2 (2.3)	1 (1.2)	1 (1.22)	1 (1.7)	
5	2 (0.6)	1 (1.1)	0 (0.0)	0 (0.0)	1 (1.7)	
6	1 (0.3)	1 (1.1)	0 (0.0)	0 (0.0)	0 (0.0)	0.59

Values are median (p25 p75) for continuous values and number (frequencies) for categorical values.

*AHRR*, Aryl-hydrocarbon receptor repressor. NSCLC, non-small-cell lung carcinoma.

^a^ p-values (two-sided) were calculated with Kruskal Wallis test for continuous values and Pearsons X^2^-test for categorical values.

^b^ Pack-years corresponding to the consumption of 20 cigarettes per day for 1 year.

^c^ First oncological treatment for lung cancer (after surgery). Surgery are performed at patients with TMN stage I and II and <5% of patients with TMN IIIa.

^d^ No platin.

^e^ ALKI/EGFR-mutation.

^f^ Other than surgery.

^g^ Within the follow-up period of the study.

As shown previously [21] AHRR methylation extent differed with cumulative smoking; the median methylation extent was 58.0% for pack-years< = 20 (interquartile range (IQR): 56.6–60.2), 56.0% pack-years >20–40 (54.7–57.1) and 55.7% for pack-years >40–60 (54.6–56.6) and 55.7% for pack-years >60 (54.3–56.6) (S1 Fig); and with smoking status; never smokers 60.2% (58.4–61.8), former smokers 56.6% (55.7–57.8) and current smokers 55.1% (54.1–56.0) (S2 Fig). Further, the expected inverse correlation between cumulative smoking and AHRR methylation was observed, as methylation (%) decreased (coefficient -0.03; 95 CI, -0.04- -0.02, p = 8.6x10^-15^) for every pack-year) and (coefficient -0.06 (-0.07- -0.04), 7.3x10-10) for every smoking-year, in the crude model (Table 2).

**Table 2:** Association between *AHRR* (cg05575921) methylation extent, smoking status, cumulative smoking (pack-years), smoking duration and cessation (years) among 465 patients with lung cancer.

	Coefficient (95% CI)	Age and sex-adjusted coefficient (95% CI)	Multivariable adjusted ^a^ coefficient (95% CI)
**Smoking status**			
Never	1.00	1.00	1.00
Former	-3.18 (-3.87- -2.49)	-3.14 (-3.84- -2.45)	-3.09 (-3.86- -2.33)
Current	-4.91 (-5.62- -4.20)	-4.87 (-5.58- -4.15)	-4.66 (-5.4- -3.86)
**Cumulative smoking, pack-years**	-0.03 (-0.04- -0.02)	-0.03 (-0.04 - -0.02)	-0.03 (-0.03- -0.02),
p-value	8.6x10^-15^	2.6x10^-15^	1.8x10^-11^
**Smoking duration, years**	-0.06 (-0.07- -0.04)	-0.09 (-0.11- -0.07)	-0.08 (-0.10- -0.06)
p-value	7.3x10^-10^	1.4x10^-19^	3.4x10^-15^
**Time since smoking cessation, years**	0.07 (0.06–0.09)	0.07 (0.05–0.09)	0.09 (0.07–0.11)
p-value	7.3x10^-13^	1.7x10^-11^	2.3x10^-18^

*AHRR*, Aryl-hydrocarbon receptor repressor. CI, confidence interval.

*A priori* potential confounders were selected, and included in models 1) A crude model, 2) A model, additionally adjusted for age at lung cancer diagnosis and sex. 3) ^a^A model, additionally adjusted for body mass index (kg/m^2^), ethnicity (European/others), TNM Classification of Malignant Tumors (TNM) (Stage I-IIII), histology of lung cancer (small cell lung cancer, adenocarcinoma, squamous-cell carcinoma, other non-small-cell lung carcinoma (NSCLC), performance status (0–3)…

Compared to individuals with cumulative smoking ≤20 pack-years, reduced survival seemed stronger among individuals with cumulative smoking >60 pack-years (HR: 1.48; 95% CI: 1.05–2.09) (Table 3). We found association with poorer performance status (p-trend = 1.1x10^-31^) and advanced TNM-status (p-trend = 2.2x10^-32^) and reduced survival (S3 Table). For performance status, which displayed the strongest association with reduced survival, the smoking plus adjusted hazard ratio for the poorest versus the best performance status was 7.48 (95% CI; 4.67–12.00).

**Table 3:** Association between cumulative smoking (pack-years) and reduced survival (from all-cause mortality) among 465 patients with lung cancer.

Cumulative smoking, pack-years, categorical variable	Crude hazard ratio for death (95% CI)	Age and sex-adjusted hazard ratio for death (95% CI)	Multivariable adjusted ^a^ hazard ratio for death (95% CI)
≤20	1.00	1.00	1.00
20–40	1.20 (0.89–1.60)	1.28 (0.94–1.71)	1.54 (1.09–2.13)
40–60	1.04 (0.78–1.39)	1.02 (0.78–1.36)	1.06 (0.75–1.49)
>60	1.48 (1.05–2.09)	1.41 (1.00–1.99)	1.29 (0.88–1.92)

CI, confidence interval.

*A priori* potential confounders were selected, and included in models 1) A crude model, 2) A model, additionally adjusted for age at lung cancer diagnosis and sex. 3) ^a^A model, additionally adjusted for body mass index (kg/m^2^), ethnicity (European/others), TNM Classification of Malignant Tumors (TNM) (Stage I-IIII), histology of lung cancer (small cell lung cancer, adenocarcinoma, squamous-cell carcinoma, other non-small-cell lung carcinoma (NSCLC), performance status (0–3).

Compared to patients in the highest AHRR methylation quartile there was no association with survival for individuals in the lowest quartile (1.00; 0.75–1.32) or the lowest decile (1.18; 0.67–2.09) versus the highest quartile or decile. In continuous models using AHRR methylation (per 1%) as exposure, results were similar (Table 4 and S6 Table).

**Table 4:** Association between *AHRR* (cg05575921) methylation extent (%) and reduced survival (from all-cause mortality) among 465 patients with lung cancer.

	Crude hazard ratio for death (95% CI)	Age and sex-adjusted hazard ratio for death (95% CI)	Multivariable adjusted ^a^hazard ratio for death (95% CI)	Smoking plus adjusted ^b^hazard ratio for death (95% CI)
***AHRR* (cg05575921) methylation extent (%), categorical variable**	n = 465	n = 465	n = 417	n = 410
57.4–63.4 (Highest)	1.00	1.00	1.00	1.00
56.0–57.4	0.87 (0.65–1.16)	0.84 (0.63–1.12)	1.01 (0.74–1.39)	0.86 (0.61–1.22)
54.9–56.0	1.03 (0.78–1.35)	1.02 (0.77–1.35)	1.09 (0.80–1.49)	0.92 (0.65–1.31)
50.0–54.9 (lowest)	1.00 (0.75–1.32)	0.99 (0.75–1.32)	1.16 (0.84–1.61)	1.03 (0.70–1.52)
p-trend				0.46
***AHRR* (cg05575921) methylation extent (%), continuous variable**	1.00 (0.95–1.04)	1.00 (0.95–1.05)	0.97 (0.92–1.02)	0.98 (0.91–1.05)
p-trend				0.40

*AHRR*, Aryl-hydrocarbon receptor repressor. CI, confidence interval.

*A priori* potential confounders were selected, and included in models 1) A crude model, 2) A model, additionally adjusted for age at lung cancer diagnosis and sex. 3) ^a^A model, additionally adjusted for body mass index (kg/m^2^), ethnicity (European/others), TNM Classification of Malignant Tumors (TNM) (Stage I-IIII), histology of lung cancer (small cell lung cancer, adenocarcinoma, squamous-cell carcinoma, other non-small-cell lung carcinoma (NSCLC), performance status (0–3), 4) ^b^A model additionally adjusted for smoking status (never/former/current smoker) and cumulative smoking (defined as 20 cigarettes/day per year, calculated from smoking intensity (number of cigarettes a day) and smoking duration (years).

In addition, we observed no associations after stratification by specific causes of death; lung cancer, cardiac disease, respiratory disease or other cancer (S7 Table).

In order to further investigate the lack of association between AHRR methylation extent and reduced survival in lung cancer patients, we also analyzed the association in individuals without lung cancer (Table 5). After multivariable adjustments including age, sex, ethnicity, body mass index and cumulative smoking, hazard ratio for death was 1.49 (95% CI; 1.16–1.93) for the lowest quantile of AHRR methylation extent versus the highest. There was no interaction between lung cancer status and AHRR methylation with survival.

**Table 5:** Association between *AHRR* (cg05575921) methylation extent in quantiles (%) and reduced survival (from all-cause mortality) in 461 matched individuals without lung cancer.

*AHRR* (cg05575921) methylation extent (%), categorical variable	Number	Crude hazard ratio for death (95% CI)	Age and sex-adjusted hazard ratio for death (95% CI)	Multivariable adjusted ^a^hazard ratio for death (95% CI)	Smoking plus adjusted ^b^hazard ratio for death (95% CI)
57.4–63.4 (Highest)	131	1.00	1.00	1.00	1.00
56.0–57.4	11	0.92 (0.45–1.89)	1.61 (0.78–3.33)	1.60 (0.77–3.32)	1.60 (0.77–3.32)
54.9–56.0	36	1.12 (0.74–1.68)	1.27 (0.84–1.91)	1.19 (0.78–1.82)	1.15 (0.75–1.76)
50.0–54.9 (Lowest)	283	1.10 (0.87–1.40)	1.58 (1.24–2.01)	1.59 (1.24–2.04)	1.49 (1.16–1.93)
**p-trend**					0.003
p-interaction*		0.96	0.89	0.99	0.87

*AHRR*, Aryl-hydrocarbon receptor repressor, CI, confidence interval.

*A priori* potential confounders available for populations without lung cancer were selected, and included in models 1) A crude model, 2) A model, additionally adjusted for age and sex. 3) ^a^A model, additionally adjusted for body mass index (kg/m^2^), ethnicity (European/others) 4) ^b^A model additionally adjusted for cumulative smoking (defined as 20 cigarettes/day per year, calculated from smoking intensity (number of cigarettes a day) and smoking duration (years).

* Results from the interaction test, including an introducing of *AHRR* (cg05575921) methylation extent X lung cancer status interaction term into the Cox model, to compare hazard ratios in Table 4 with hazard ratios in Table 5.

## Discussion

In this study of 465 lung cancer cases, we observed no association between *AHRR* (cg05575921) methylation and survival.

With 81.7% mortality after a median follow up time of 332 days, the overall prognosis for the patient was as expected for lung cancer [27]. Further, since reduced survival was strongly associated with high cumulative tobacco consumption, poor performance status, and with advanced TNM we expect data to be in line with the usual finding. Also, the measurement of *AHRR* methylation was precise enough to pick up the expected association with cumulative smoking. Therefore, since both exposure and outcome variables were presumably accurately measured and we observed the expected association between *AHRR* methylation and reduced survival among individuals without lung cancer, we assume that the lack of association is correct, and not caused by measurement or registration errors.

We speculate that the lack of association could be due to individual threshold phenomena, where smoking can only reduce *AHRR* methylation to a certain low level in each individual, and the level of this threshold is not determined by factors associated with reduced survival. Having reached this threshold, more intensive smoking reduces survival, but will not reduce methylation further as suggested by the very narrow range of *AHRR* methylation in the lung cancer patients compared with the matched individuals from the general population without lung cancer. Further, since *AHRR* methylation only reverts very slowly over decades after smoking cessation [6], the prognostic benefit from quitting within years before blood sampling, cannot be monitored by measuring *AHRR* methylation, especially in cohorts with poor short-term prognosis, i.e. lung cancer patients. Further, we found that *AHRR* hypomethylation behaves different on reduced survival in lung cancer patients than in individuals without lung cancer. Along with prior studies [15,17,19] showing association between *AHRR* hypomethylation and increased lung cancer risk, these findings suggest that smoking-associated methylation changes are more involved in initiation than progression of lung cancer.

Although *AHRR* methylation might be useful in assessing lung cancer risk in a population of smokers [19], our results suggest that it cannot identify lung cancer patients with poor prognosis independently of other commonly used prognostic factors.

Strengths of this study was the prospective design with detailed information on many covariates, complete follow-up, and no misclassification of vital status in Denmark. Lung cancer diagnosis was based on pathology confirmation by a trained pathologist.

Some limitations must, however, be acknowledged. 16 (3,4%) patients had >30 days from date of blood sample and date of diagnosis. However, these represented a non-differential sample according to reduced survival. Possible changes in *AHRR* methylation during follow-up could not been taken into account. Also, we do not have information on the exposure of passive smoking. This would have been relevant to include in our analyses since *AHRR* hypomethylation has also been found in those exposed to passive smoke [28]. Since it is also associated with reduced survival [29], inclusion of passive smoking in the analyses would probably have weakened the association between smoking and *AHRR* hypomethylation, but likely not the association between *AHRR* hypomethylation and reduced survival.

## Conclusion

AHRR (cg05575921) methylation cannot be used to predict smoking mediated reduced survival in lung cancer patients.

## Supporting information

S1 Table Baseline characteristics of 461 individuals without lung cancer by leukocyte DNA methylation of *AHRR* (cg05575921), quantiles. Values are median (p25 p75) for continuous values and number (frequencies) for categorical values. *AHRR*, Aryl-hydrocarbon receptor repressor.
Matching:
Lung cancer free individuals were matched 1:1 with the lung cancer patients using categories of body mass index (< = 18, >18 to < = 25, >25 to < = 30, >30 to < = 40, > 40 kg/m^2^), cumulative smoking in 10-pack year groups, age in 10-year groups, and sex.
^a^ p-values (two-sided) were calculated with Kruskal Wallis test for continuous values and Pearsons X^2^-test for categorical values.
^b^ Pack-years corresponding to the consumption of 20 cigarettes per day for 1 year.
(DOCX)

S2 Table Association between *AHRR* (cg05575921) methylation extent, smoking status cumulative smoking (pack-years), smoking duration (years) and smoking cessation among 461 individuals without lung cancer. *AHRR*, Aryl-hydrocarbon receptor repressor. CI, confidence interval.
*A priori* potential confounders available for populations without lung cancer were selected, and included in models 1) A crude model, 2) A model, additionally adjusted for age and sex. 3) ^a^A model, additionally adjusted for body mass index (kg/m^2^) and ethnicity (European/others).
(DOCX)

S3 Table Association between performance status and TNM Classification of Malignant Tumours (TNM) and reduced survival (from all-cause mortality) among 465 patients with lung cancer. AHRR, Aryl-hydrocarbon receptor repressor CI, confidence interval.
A priori potential confounders were selected, and included in models 1) A crude model, 2) A model adjusted for age at lung cancer diagnosis and sex. 3) ^a^A model, additionally adjusted for body mass index (kg/m2), ethnicity (European/others), histology of lung cancer (small cell lung cancer, adenocarcinoma, squamous-cell carcinoma, other non-small-cell lung carcinoma (NSCLC) and (if not the exposure variable) ECOG performance status (0–3), TNM Classification of Malignant Tumors (TNM) (Stage I-IIII). 4) ^b^ A model, additionally adjusted for cumulative smoking (defined as 20 cigarettes/day per year, calculated from smoking intensity (number of cigarettes a day) and smoking duration (years).
(DOCX)

S4 Table Association between oncological treatment for lung cancer, recurrence of lung cancer, total lines of treatment and reduced survival (from all-cause mortality) among 465 patients with lung cancer. CI, confidence interval.
^a^ Include platinum-based chemotherapy, combination and monotherapy, targeted therapy (ALKi/EGFR-mutation), immunotherapy, no oncological treatment other than surgery.
^b^ Within the follow-up period of the study.
A priori potential confounders were selected, and included in models 1) A crude model, 2) A model, additionally adjusted for age at lung cancer diagnosis and sex. 3) ^c^A model, additionally adjusted for body mass index (kg/m^2^), ethnicity (European/others), TNM Classification of Malignant Tumors (TNM) (Stage I-IIII), histology of lung cancer (small cell lung cancer, adenocarcinoma, squamous-cell carcinoma, other NSCLC), ECOG performance status (0–3), 4) ^d^A model additionally adjusted for smoking status (never/former/current smoker) and cumulative smoking (defined as 20 cigarettes/day per year, calculated from smoking intensity (number of cigarettes a day) and smoking duration (years).
(DOCX)

S5 Table Association between *AHRR* (cg05575921) methylation extent and reduced survival (from all-cause mortality) by oncological treatment for lung cancer, recurrence of lung cancer and total lines of treatment among 465 patients with lung cancer. *AHRR*, Aryl-hydrocarbon receptor repressor CI, confidence interval.
^a^ Include platinium-based chemotherapy, combination and monotherapy, targeted therapy (ALKI/EGFR-mutation), immunotherapy, no oncological treatment other than surgery.
*A priori* potential confounders were selected, and included in models 1) A crude model, 2) A model, additionally adjusted for age at lung cancer diagnosis and sex. 3) ^b^A model, additionally adjusted for body mass index (kg/m^2^), ethnicity (European/others), TNM Classification of Malignant Tumors (TNM) (Stage I-IIII), histology of lung cancer (small cell lung cancer, adenocarcinoma, squamous-cell carcinoma, other non-small-cell lung carcinoma (NSCLC), ECOG performance status (0–3), 4) ^c^A model additionally adjusted for smoking status (never/former/current smoker) and cumulative smoking (defined as 20 cigarettes/day per year, calculated from smoking intensity (number of cigarettes a day) and smoking duration (years).
(DOCX)

S6 Table Association between *AHRR* (cg05575921) methylation extent in deciles (%) and reduced survival (from all-cause mortality) among 465 patients with lung cancer. *AHRR*, Aryl-hydrocarbon receptor repressor, CI, confidence interval.
*A priori* potential confounders were selected, and included in models 1) A crude model, 2) A model, additionally adjusted for age at lung cancer diagnosis and sex. 3) ^a^A model, additionally adjusted for body mass index (kg/m^2^), ethnicity (European/others), TNM Classification of Malignant Tumors (TNM) (Stage I-IIII), histology of lung cancer (small cell lung cancer, adenocarcinoma, squamous-cell carcinoma, other non-small-cell lung carcinoma (NSCLC), ECOG performance status (0–3), 4) ^b^A model additionally adjusted for smoking status (never/former/current smoker) and cumulative smoking (defined as 20 cigarettes/day per year, calculated from smoking intensity (number of cigarettes a day) and smoking duration (years).
(DOCX)

S7 Table Association between by *AHRR* (cg05575921) methylation extent and reduced survival by registered cause of death^a^ (lung cancer, cardiac disease, respiratory disease and other cancers) among 385 patients with lung cancer. *AHRR*, Aryl-hydrocarbon receptor repressor CI, confidence interval.
^a^ One individual is registered with up till nine causes of death, and might be included in analyzes with several causes of death.
*A priori* potential confounders were selected, and included in models 1) A crude model, 2) A model, adjusted for age at lung cancer diagnosis and sex. 3) ^b^A model, additionally adjusted for body mass index (kg/m^2^), ethnicity (European/others), TNM Classification of Malignant Tumors (TNM) (Stage I-IIII), histology of lung cancer (small cell lung cancer, adenocarcinoma, squamous-cell carcinoma, other non-small-cell lung carcinoma (NSCLC), ECOG performance status (0–3) 4) ^c^A model additionally adjusted for smoking status (never/former/current smoker) and cumulative smoking (defined as 20 cigarettes/day per year, calculated from smoking intensity (number of cigarettes a day) and smoking duration (years).
(DOCX)

S1 Fig Cumulative smoking as a function of *AHRR* (cg05575921) methylation extent (%) among former and current smokers among patients with lung cancer. **Values are median (p25 p75).** *AHRR*, Aryl-hydrocarbon receptor repressor
p-values (two-sided) were calculated with Pearsons X^2^-test for categorical values.
(TIF)

S2 Fig Smoking status as a function of *AHRR* (cg05575921) methylation extent (%) among patients with lung cancer. **Values are median (p25 p75)**.
*AHRR*, Aryl-hydrocarbon receptor repressor
p-values (two-sided) were calculated with Pearsons X^2^-test for categorical values.
(TIF)

S3 Fig Kaplan-Meier plot of all-cause survival by *AHRR* (cg05575921) methylation extent in quantiles (%) among 465 patients with lung cancer. *AHRR*, Aryl-hydrocarbon receptor repressor
1st: Highest quantiles of *AHRR* (cg05575921) methylation extent.
4st: Lowest quantiles of *AHRR* (cg05575921) methylation extent.
(TIF)
