# Supplementary material for: AHRR (cg05575921) methylation extent of leukocyte DNA and lung cancer survival
Source: PLoS One. 2019 Feb 7;14(2):e0211745. doi: 10.1371/journal.pone.0211745 (PMC6366765; doi:10.1371/journal.pone.0211745)
Supplement: S1 Table — Values are median (p25 p75) for continuous values and number (frequencies) for categorical values. AHRR, Aryl-hydrocarbon receptor repressor. Matching: Lung cancer free individuals were matched 1:1 with the lung cancer patients using categories of body mass index (< = 18, >18 to < = 25, >25 to < = 30, >30 to < = 40, > 40 kg/m2), cumulative smoking in 10-pack year groups, age in 10-year groups, and sex. a p-values (two-sided) were calculated with Kruskal Wallis test for continuous values and Pearsons X2-test for categorical values. b Pack-years corresponding to the consumption of 20 cigarettes per day for 1 year. (DOCX) [file pone.0211745.s001.docx]

**S1 Table. Baseline characteristics of 461 individuals without lung cancer by leukocyte DNA methylation of *AHRR* (cg05575921), quantiles.**

|  | Total | **Leukocyte DNA methylation of *AHRR* (cg05575921), quantiles** | | | | |
| --- | --- | --- | --- | --- | --- | --- |
|  |  | **1^st^ (highest)**  **57.4-63.4%** | **2^nd^**  **56.0-57.4%** | **3^rd^**  **54.9-56.0%** | **4^th^ (lowest)**  **50.0-54.9%** | **p-value^a^** |
| **Total (%)** | 461 | 131 (28.4) | 11 (2.4) | 36 (7.8) | 283 (61.4 | - |
| **Age years** | 67.2 (62.3-72.9) | 71.4 (64.8-78.4) | 64.8 (58.1-73.8) | 68.4 (64.2-74.6) | 67.2 (61.3-72.9) | 0.0007 |
| ***AHRR* (cg05575921) methylation extent, %** | 53.0 (48.0-58.0) | 62.0 (59.0-65.0) | 57.0 (56.5-57.0) | 55.0 (55.0-55.5) | 49.0 (46.0-52.0) | 0.0001 |
| **Sex**  **Men**  **Women** | 267 (57.9)  194 (42.1) | 76 (58.0)  55 (42.0) | 3 (27.3)  8 (72.7) | 23 (63.9)  13 (36.1) | 165 (58.3)  118 (41.7) | 0.19 |
| **Ethnicity,**  **European**  **Others** | 456 (98.9)  5 (1.1) | 130 (99.3)  1 (0.76) | 11 (100.0)  0 (0.0) | 33 (91.7)  3 (8.3) | 282 (99.7)  1 (0.35) | 0.0002 |
| **Body mass index (kg/m^2^)**  **<18.5**  **18.5-24.9**  **25.0-29.9**  **≥30.0** | 34 (7.8)  235 (51.0)  155 (33.6)  37 (8.0) | 4 (3.1)  52 (39.7)  56 (42.8)  19 (14.5) | 1 (9.1)  6 (54.6)  3 (27.3)  1 (9.1) | 2 (5.6)  14 (38.9)  15 (41.7)  5 (13.9) | 27 (9.5)  163 (57.6)  81 (28.6)  12 (4.2) | 0.0002 |
| **Smoking status**  **Never**  **Former (>6 months ago)**  **Current** | 1 (0.2)  178 (38.6)  282 (61.2) | 0 (0.0)  99 (75.6)  32 (24.4) | 1 (9.1)  7 (63.6)  3 (27.3) | 0 (0.0)  20 (55.6)  16 (44.4) | 0 (0.0)  52 (18.4)  231 (81.6) | 7.3x10^-35^ |
| **Cumulative smoking^b^, pack-years** | 43.0 (28.0-54.0) | 32.0 (15.0-48.4) | 29.5 (5.6-40.5) | 43.9 (29.5-57.4) | 46.1 (31.2-55.4) | 0.0001 |
| **Smoking duration, years** | 43.0 (31.0-51.0) | 30.0 (20.0-45.5) | 40.0 (25.0-45.0) | 40.0 (31.0-50.0) | 46.0 (40.0-53.0) | 0.0001 |
| **Time since smoking cessation, years** | 15.7 (7.0-27.6) | 20.4 (9.7-33.6) | 5.8 (2.1-26.1) | 12.9 (7.6-19.0) | 11.4 (6.0-17.4) | 0.0012 |
| **Death**  **No**  **Yes** | 467 (94.8)  24 (5.2) | 126 (96.2)  5 (3.8) | 11 (100.0)  0 (0.0) | 33 (91.7)  3 (8.3) | 267 (94.4)  16 (5.7) | 0.58 |

Values are median (p25 p75) for continuous values and number (frequencies) for categorical values.

*AHRR*, Aryl-hydrocarbon receptor repressor.

Matching:

Lung cancer free individuals were matched 1:1 with the lung cancer patients using categories of body mass index (<=18, >18 to <=25, >25 to <= 30, >30 to <=40, > 40 kg/m^2^), cumulative smoking in 10-pack year groups, age in 10-year groups, and sex.

^a^ p-values (two-sided) were calculated with Kruskal Wallis test for continuous values and Pearsons X^2^-test for categorical values.

^b^ Pack-years corresponding to the consumption of 20 cigarettes per day for 1 year.
