# Supplementary material for: AHRR (cg05575921) methylation extent of leukocyte DNA and lung cancer survival
Source: PLoS One. 2019 Feb 7;14(2):e0211745. doi: 10.1371/journal.pone.0211745 (PMC6366765; doi:10.1371/journal.pone.0211745)
Supplement: S2 Table — AHRR, Aryl-hydrocarbon receptor repressor. CI, confidence interval. A priori potential confounders available for populations without lung cancer were selected, and included in models 1) A crude model, 2) A model, additionally adjusted for age and sex. 3) aA model, additionally adjusted for body mass index (kg/m2) and ethnicity (European/others). (DOCX) [file pone.0211745.s002.docx]

**S2 Table. Association between *AHRR* (cg05575921) methylation extent, smoking status cumulative smoking (pack-years), smoking duration (years) and smoking cessation among 461 individuals without lung cancer.**

|  | **Coefficient (95% CI)** | **Age and sex-adjusted coefficient (95% CI)** | **Multivariable adjusted ^a^**  **Coefficient (95% CI)** |
| --- | --- | --- | --- |
| **Smoking status**  **Never**  **Former**  **Current** | 1.00  1.63 (-11.6- -14.9)  -6.7 (-20.0-6.50) | 1.00  2.22 (-10.96-15.4)  -5.79 (18.97-7.38) | 1.00  0.68 (-12.62-13.98)  -6.88 (-20.15-6.39) |
| **Cumulative smoking, pack-years, p-value** | -0.05 (-0.08- -0.03), 0.0001 | -0.06 (-0.09- -0.04), 8.9x10^-6^ | -0.07 (-0.10- -0.04), 4,1x10^-7^ |
| **Smoking duration, years, p-value** | -0.18 (-0.22- -0.13), 1.9x10^-13^ | -0.25 (-0.30- -0.20), 3.9x10^-23^ | -0.23 (-0.28- -0.19), 1.5x10^-20^ |
| **Time since smoking cessation, years, p-value** | 0.19 (0.12-0.26), 4.7x10^-7^ | 0.21 (0.14-0.29), 8.7x10^-8^ | 0.22 (0.14-0.29), 4.3x10^-8^ |

CI, confidence interval.

*A priori* potential confounders available for populations without lung cancer were selected, and included in models 1) A crude model, 2) A model, additionally adjusted for age and sex. 3) ^a^A model, additionally adjusted for body mass index (kg/m^2^) and ethnicity (European/others)
