# Supplementary material for: AHRR (cg05575921) methylation extent of leukocyte DNA and lung cancer survival
Source: PLoS One. 2019 Feb 7;14(2):e0211745. doi: 10.1371/journal.pone.0211745 (PMC6366765; doi:10.1371/journal.pone.0211745)
Supplement: S3 Table — AHRR, Aryl-hydrocarbon receptor repressor CI, confidence interval. A priori potential confounders were selected, and included in models 1) A crude model, 2) A model adjusted for age at lung cancer diagnosis and sex. 3) aA model, additionally adjusted for body mass index (kg/m2), ethnicity (European/others), histology of lung cancer (small cell lung cancer, adenocarcinoma, squamous-cell carcinoma, other non-small-cell lung carcinoma (NSCLC) and (if not the exposure variable) ECOG performance status (0–3), TNM Classification of Malignant Tumors (TNM) (Stage I-IIII). 4) b A model, additionally adjusted for cumulative smoking (defined as 20 cigarettes/day per year, calculated from smoking intensity (number of cigarettes a day) and smoking duration (years). (DOCX) [file pone.0211745.s003.docx]

**S3 Table. Association between performance status and TNM Classification of Malignant Tumours (TNM) and reduced survival (from all-cause mortality) among 465 patients with lung cancer.**

|  | **Number** | **Crude hazard ratio for death (95% CI)** | **Age and sex-adjusted**  **hazard ratio for death (95% CI)** | **Multivariable adjusted ^a^**  **hazard ratio for death (95% CI)** | **Smoking plus adjusted ^b^**  **hazard ratio for death (95% CI)** |
| --- | --- | --- | --- | --- | --- |
| **Performance status**  **0**  **1**  **2**  **3**  **p-trend**  **TNM Classification of Malignant Tumours (TNM)**  **Stage I**  **Stage II**  **Stage III**  **Stage IIII**  **p-trend** | 158  186  81  35  26  33  163  242 | 1.00  1.81 (1.41-2.31)  3.30 (2.25-4.45)  9.32 (6.24-13.92)  1.00  2.27 (1.03-4.99)  3.31 (1.68-6.52)  7.98 (4.08-15.60) | 1.00  1.80 (1.40-2.32)  3.28 (2.42-4.44)  9.55 (6.35-14.40)  1.00  2.20 (1.00-4.83)  3.21 (1.63-6.34)  8.00 (4.08-15.6) | 1.00  1.64 (1.25-2.13)  2.67 (1.94-3.68)  8.06 (5.12-12.70)  1.00  1.60 (0.72-3.57)  2.68 (1.35-5.35)  6.16 (3.10-12.2) | 1.00  1.53 (1.17-2.02)  2.45 (1.76-3.43)  7.48 (4.67-12.00)  1.1x10^-31^  1.00  1.51 80.66-3.54)  2.59 (1.30-5.17)  6.20 (3.11-12.40)  2.2x10^-32^ |

*AHRR*, Aryl-hydrocarbon receptor repressor CI, confidence interval.

*A priori* potential confounders were selected, and included in models 1) A crude model, 2) A model adjusted for age at lung cancer diagnosis and sex. 3) ^a^A model, additionally adjusted for body mass index (kg/m2), ethnicity (European/others), histology of lung cancer (small cell lung cancer, adenocarcinoma, squamous-cell carcinoma, other non-small-cell lung carcinoma (NSCLC) and (if not the exposure variable) performance status, TNM Classification of Malignant Tumors (TNM) (Stage I-IIII). 4) ^b^ A model, additionally adjusted for cumulative smoking (defined as 20 cigarettes/day per year, calculated from smoking intensity (number of cigarettes a day) and smoking duration (years).
