# Supplementary material for: AHRR (cg05575921) methylation extent of leukocyte DNA and lung cancer survival
Source: PLoS One. 2019 Feb 7;14(2):e0211745. doi: 10.1371/journal.pone.0211745 (PMC6366765; doi:10.1371/journal.pone.0211745)
Supplement: S4 Table — CI, confidence interval. a Include platinum-based chemotherapy, combination and monotherapy, targeted therapy (ALKi/EGFR-mutation), immunotherapy, no oncological treatment other than surgery. b Within the follow-up period of the study. A priori potential confounders were selected, and included in models 1) A crude model, 2) A model, additionally adjusted for age at lung cancer diagnosis and sex. 3) cA model, additionally adjusted for body mass index (kg/m2), ethnicity (European/others), TNM Classification of Malignant Tumors (TNM) (Stage I-IIII), histology of lung cancer (small cell lung cancer, adenocarcinoma, squamous-cell carcinoma, other NSCLC), ECOG performance status (0–3), 4) dA model additionally adjusted for smoking status (never/former/current smoker) and cumulative smoking (defined as 20 cigarettes/day per year, calculated from smoking intensity (number of cigarettes a day) and smoking duration (years). (DOCX) [file pone.0211745.s004.docx]

**S4 Table. Association between oncological treatment for lung cancer, recurrence of lung cancer and total lines of treatment and reduced survival (from all-cause mortality) among 465 patients with lung cancer.**

|  | **Number** | **Crude hazard ratio for death (95% CI)** | **Age and sex-adjusted hazard ratio for death (95% CI)** | **Multivariable adjusted ^c^**  **hazard ratio for death (95% CI)** | **Smoking plus adjusted ^d^**  **hazard ratio for death (95% CI)** |
| --- | --- | --- | --- | --- | --- |
| **Oncological treatment for lung cancer^a^**  **No**  **Yes**  **Recurrence of lung cancer^b^**  **No**  **Yes**  **Total lines of treatment**  **1**  **>1** | 151  314  368  97  203  111 | 1.00  1.08 (0.87-1.35)  1.00  0.74 (0.58-0.95)  1.00  0.73 (0.60-0.90) | 1.00  1.09 (0.88-1.36)  1.00  0.77 (0.60-0.99)  1.00  0.76 (0.62-0.94) | 1.00  0.87 (0.66-1.13)  1.00  0.80 (0.61-1.06)  1.00  0.91 (0.73-1.14) | 1.00  0.86 (0.66-1.13)  1.00  0.76 (0.58-1.01)  1.00  0.87 (0.69-1.10) |

CI, confidence interval.

^a^ Include platinium-based chemotherapy, combination and monotherapy, targeted therapy (ALKI/EGFR-mutation), immunotherapy, no oncological treatment other than surgery.

^b^ Within the follow-up period of the study.

*A priori* potential confounders were selected, and included in models 1) A crude model, 2) A model, additionally adjusted for age at lung cancer diagnosis and sex. 3) ^c^A model, additionally adjusted for body mass index (kg/m2) , ethnicity (European/others), TNM Classification of Malignant Tumors (TNM) (Stage I-IIII), histology of lung cancer (small cell lung cancer, adenocarcinoma, squamous-cell carcinoma, other NSCLC), performance status (0-4), 4) ^d^A model additionally adjusted for smoking status (never/former/current smoker) and cumulative smoking (defined as 20 cigarettes/day per year, calculated from smoking intensity (number of cigarettes a day) and smoking duration (years).
