# Supplementary material for: AHRR (cg05575921) methylation extent of leukocyte DNA and lung cancer survival
Source: PLoS One. 2019 Feb 7;14(2):e0211745. doi: 10.1371/journal.pone.0211745 (PMC6366765; doi:10.1371/journal.pone.0211745)
Supplement: S5 Table — AHRR, Aryl-hydrocarbon receptor repressor CI, confidence interval. a Include platinium-based chemotherapy, combination and monotherapy, targeted therapy (ALKI/EGFR-mutation), immunotherapy, no oncological treatment other than surgery. A priori potential confounders were selected, and included in models 1) A crude model, 2) A model, additionally adjusted for age at lung cancer diagnosis and sex. 3) bA model, additionally adjusted for body mass index (kg/m2), ethnicity (European/others), TNM Classification of Malignant Tumors (TNM) (Stage I-IIII), histology of lung cancer (small cell lung cancer, adenocarcinoma, squamous-cell carcinoma, other non-small-cell lung carcinoma (NSCLC), ECOG performance status (0–3), 4) cA model additionally adjusted for smoking status (never/former/current smoker) and cumulative smoking (defined as 20 cigarettes/day per year, calculated from smoking intensity (number of cigarettes a day) and smoking duration (years). (DOCX) [file pone.0211745.s005.docx]

**S5 Table. Association between *AHRR* methylation extent and reduced survival (from all-cause mortality) by oncological treatment for lung cancer, recurrence of lung cancer and total lines of treatment among 465 patients with lung cancer.**

|  | **Number** | **Methylation extent, median (interquartile range)** | **Crude hazard ratio for death (95% CI)** | **Age and sex-adjusted**  **hazard ratio for death (95% CI)** | **Multivariable adjusted ^b^**  **hazard ratio for death (95% CI)** | **Smoking plus adjusted ^c^**  **hazard ratio for death (95% CI)** |
| --- | --- | --- | --- | --- | --- | --- |
| **Oncological treatment for lung cancer^a^**  **No**  **57.4-63.4 (Highest)**  **56.0-57.4**  **54.9-56.0**  **50.0-54.9 (Lowest)**  **p-trend**  **Yes**  **57.4-63.4 (Highest)**  **56.0-57.4**  **54.9-56.0**  **50.0-54.9 (Lowest)**  **p-trend**  **p-interaction**  **Recurrence of lung cancer**  **No**  **57.4-63.4 (Highest)**  **56.0-57.4**  **54.9-56.0**  **50.0-54.9 (Lowest)**  **p-trend**  **Yes**  **57.4-63.4 (Highest)**  **56.0-57.4**  **54.9-56.0**  **50.0-54.9 (Lowest)**  **p-trend**  **p-interaction**  **Total lines of treatment**  **1**  **57.4-63.4 (Highest)**  **56.0-57.4**  **54.9-56.0**  **50.0-54.9 (Lowest)**  **p-trend**  **>1**  **57.4-63.4 (Highest)**  **56.0-57.4**  **54.9-56.0**  **50.0-54.9 (Lowest)**  **p-interaction** | 151  314  368  97  203  111 | 55.5 (54.3-56.9)  56.35 (55.1-57.5)  56.0 (54.7-57.45)  56.1 (55.1-57.1)  55.5 (54.3-56.9)  56.35 (55.1-57.5) | 1.00  0.65 (0.36-1.19)  0.73 (0.44-1.23)  0.76 (0.48-1.22)  1.00  0.95 (0.68-1.33)  1.19 (0.86-1.66)  1.19 (0.83-1.70)  1.00  0.85 (0.61-1.18)  0.97 (0.71-1.33)  1.05 (0.77-1.43)  1.00  1.03 (0.56-1.90)  1.44 (0.79-2.60)  0.67 (0.33-1.36)  1.00  0.90 (0.60-1.37)  1.12 (0.75-1.68)  1.44 (0.94-2.21)  1.00  0.82 (0.55-1.23)  0.97 (0.66-1.42)  0.83 (0.57-1.22) | 1.00  0.65 (0.35-1.20)  0.78 (0.46-1.35)  0.77 (0.47-1.27)  1.00  0.93 (0.66-1.29)  1.17 (0.85-1.63)  1.19 (0.83-1.71)  1.00  0.79 (0.57-1.11)  0.95 (0.69-1.31)  1.07 (0.79-1.46)  1.00  0.90 (0.47-1.73)  1.27 (0.68-2.38)  0.55 (0.26-1.14)  1.00  0.85 (0.56-1.30)  1.08 (0.72-1.62)  1.52 (0.99-2.36)  1.00  0.82 (0.54-1.23)  0.99 (0.67-1.46)  0.79 (0.54-1.16) | 1.00  0.33 (0.15-0.74)  0.57 (0.31-1.06)  0.63 (0.34-1.19)  1.00  1.18 (0.83-1.69)  1.25 (0.87-1.81)  1.22 (0.80-1.84)  1.00  0.89 (0.61-1.28)  1.06 (0.74-1.51)  1.23 (0.86-1.75)  1.00  0.99 (0.47-2.08)  1.54 (0.72-3.30)  0.58 (0.25-1.35)  1.00  1.11 (0.69-1.78)  1.31 (0.81-2.11)  1.34 (0.81-2.22)  1.00  0.81 (0.51-1.27)  0.99 (0.64-1.52)  0.86 80.54-1.36) | 1.00  0.19 (0.08-0.48)  0.45 (0.22-0.94)  0.49 (0.23-1.05)  0.32  1.00  1.04 (0.70-1.55)  1.08 (0.71-1.64)  1.08 (0.67-1.75)  0.28  0.32  1.00  0.80 (0.54-1.18)  0.97 (0.65-1.44)  1.15 (0.77-1.74)  0.17  1.00  1.31 (0.55-3.14)  2.14 (0.88-5.21)  0.80 (0.30-2.12)  0.44  0.05  1.00  1.09 (0.67-1.79)  1.19 (0.71-2.01)  1.20 (0.68-2.10)  0.08  1.00  0.73 (0.44-1.22)  0.96 (0.59-1.58)  0.86 (0.49-1.48)  0.21 |

*AHRR*, Aryl-hydrocarbon receptor repressor CI, confidence interval.

^a^ Include platinium-based chemotherapy, combination and monotherapy, targeted therapy (ALKI/EGFR-mutation), immunotherapy, no oncological treatment other than surgery.

*A priori* potential confounders were selected, and included in models 1) A crude model, 2) A model, additionally adjusted for age at lung cancer diagnosis and sex. 3) ^b^A model, additionally adjusted for body mass index (kg/m2), ethnicity (European/others), TNM Classification of Malignant Tumors (TNM) (Stage I-IIII), histology of lung cancer (small cell lung cancer, adenocarcinoma, squamous-cell carcinoma, other non-small-cell lung carcinoma (NSCLC), performance status (0-4), 4) ^c^A model additionally adjusted for smoking status (never/former/current smoker) and cumulative smoking (defined as 20 cigarettes/day per year, calculated from smoking intensity (number of cigarettes a day) and smoking duration (years).
