# Supplementary material for: AHRR (cg05575921) methylation extent of leukocyte DNA and lung cancer survival
Source: PLoS One. 2019 Feb 7;14(2):e0211745. doi: 10.1371/journal.pone.0211745 (PMC6366765; doi:10.1371/journal.pone.0211745)
Supplement: S6 Table — AHRR, Aryl-hydrocarbon receptor repressor, CI, confidence interval. A priori potential confounders were selected, and included in models 1) A crude model, 2) A model, additionally adjusted for age at lung cancer diagnosis and sex. 3) aA model, additionally adjusted for body mass index (kg/m2), ethnicity (European/others), TNM Classification of Malignant Tumors (TNM) (Stage I-IIII), histology of lung cancer (small cell lung cancer, adenocarcinoma, squamous-cell carcinoma, other non-small-cell lung carcinoma (NSCLC), ECOG performance status (0–3), 4) bA model additionally adjusted for smoking status (never/former/current smoker) and cumulative smoking (defined as 20 cigarettes/day per year, calculated from smoking intensity (number of cigarettes a day) and smoking duration (years). (DOCX) [file pone.0211745.s006.docx]

**S6 Table. Association between *AHRR* (cg05575921) methylation extent in deciles (%) and reduced survival (from all-cause mortality) among 465 patients with lung cancer**.

|  | **Number** | **Crude hazard ratio for death (95% CI)** | **Age and gender-adjusted**  **hazard ratio for death (95% CI)** | **Multivariable adjusted ^a^**  **hazard ratio for death (95% CI)** | **Smoking plus adjusted ^b^**  **hazard ratio for death (95% CI)** |
| --- | --- | --- | --- | --- | --- |
| Total number | 465 | 465 | 465 | 417 | 410 |
| ***AHRR* (cg05575921) methylation extent (%), categorical variable**  **59.2-63.4**  **58.0-59.1**  **57.2-57.8**  **56.8-57.1**  **56.1-56.6**  **55.8-56.0**  **55.2-55.7**  **54.6-55.1**  **54.0-54.4**  **50.1-53.8** | 53  43  51  51  35  56  47  36  48  45 | 1.00  0.73 (0.46-1.14)  1.13 (0.71-1.81)  0.70 (0.45-1.11)  0.87 (0.57-1.33)  0.81 (0.50-1.32)  1.02 (0.67-1.55)  0.93 (0.60-1.43)  1.14 (0.73-1.78)  0.81 (0.53-1.24) | 1.00  0.73 (0.47-1.15)  1.05 (0.66-1.68)  0.65 (0.41-1.03)  0.87 (0.57-1.32)  0.75 (0.46-1.23)  1.01 (0.66-1.54)  0.96 (0.62-1.48)  1.11 (0.71-1.74)  0.79 (0.52-1.22) | 1.00  0.98 (0.59-1.63)  1.49 (0.89-2.48)  0.91 (0.56-1.49)  1.23 (0.77-1.99)  1.00 (0.59-1.69)  1.31 (0.83-2.07)  1.13 (0.70-1.84)  1.36 (0.83-2.22)  1.26 (0.78-2.01) | 1.00  0.87 (0.50-1.50)  1.31 (0.75-2.26)  0.78 (0.45-1.36)  1.09 (0.63-1.89)  0.87 (0.48-1.56)  1.13 (0.66-1.96)  1.02 (0.58-1.80)  1.21 (0.69-2.11)  1.18 (0.67-2.09) |

*AHRR*, Aryl-hydrocarbon receptor repressor, CI, confidence interval.

*A priori* potential confounders were selected, and included in models 1) A crude model, 2) A model, additionally adjusted for age at lung cancer diagnosis and sex. 3) ^a^A model, additionally adjusted for body mass index (kg/m2) , ethnicity (European/others), TNM Classification of Malignant Tumors (TNM) (Stage I-IIII), histology of lung cancer (small cell lung cancer, adenocarcinoma, squamous-cell carcinoma, other non-small-cell lung carcinoma (NSCLC), performance status (0-4), 4) ^b^A model additionally adjusted for smoking status (never/former/current smoker) and cumulative smoking (defined as 20 cigarettes/day per year, calculated from smoking intensity (number of cigarettes a day) and smoking duration (years).
