# Supplementary material for: AHRR (cg05575921) methylation extent of leukocyte DNA and lung cancer survival
Source: PLoS One. 2019 Feb 7;14(2):e0211745. doi: 10.1371/journal.pone.0211745 (PMC6366765; doi:10.1371/journal.pone.0211745)
Supplement: S7 Table — AHRR, Aryl-hydrocarbon receptor repressor CI, confidence interval. a One individual is registered with up till nine causes of death, and might be included in analyzes with several causes of death. A priori potential confounders were selected, and included in models 1) A crude model, 2) A model, adjusted for age at lung cancer diagnosis and sex. 3) bA model, additionally adjusted for body mass index (kg/m2), ethnicity (European/others), TNM Classification of Malignant Tumors (TNM) (Stage I-IIII), histology of lung cancer (small cell lung cancer, adenocarcinoma, squamous-cell carcinoma, other non-small-cell lung carcinoma (NSCLC), ECOG performance status (0–3) 4) cA model additionally adjusted for smoking status (never/former/current smoker) and cumulative smoking (defined as 20 cigarettes/day per year, calculated from smoking intensity (number of cigarettes a day) and smoking duration (years). (DOCX) [file pone.0211745.s007.docx]

**S7 Table. Association between by *AHRR* (cg05575921) methylation extent and reduced survival by registered cause of death^a^ (lung cancer, cardiac disease, respiratory disease and other cancers) for 385 patients with lung cancer.**

| **Cause of death** | **Number (vs. number of other causes of death)^a^** | **Crude hazard ratio for death (95% CI)** | **Age and sex-adjusted hazard ratio for death (95% CI)** | **Multivariable adjusted ^b^**  **hazard ratio for death (95% CI)** | **Smoking plus adjusted ^c^**  **hazard ratio for death (95% CI)** |
| --- | --- | --- | --- | --- | --- |
| **Lung cancer**  **57.4-63.4 (Highest)**  **56.0-57.4**  **54.9-56.0**  **50.0-54.9 (Lowest)**  **p-trend**  **Cardiac disease**  **57.4-63.4 (Highest)**  **56.0-57.4**  **54.9-56.0**  **50.0-54.9 (Lowest)**  **p-trend**  **Respiratory disease**  **57.4-63.4 (Highest)**  **56.0-57.4**  **54.9-56.0**  **50.0-54.9 (Lowest)**  **p-trend**  **Other cancer**  **57.4-63.4 (Highest)**  **56.0-57.4**  **54.9-56.0**  **50.0-54.9 (Lowest)**  **p-trend** | 320 (vs. 65)  47 (vs. 338)  133 (vs. 252)  109 (vs. 276) | 1.00  1.00 (0.73-1.37)  1.00 (0.74-1.35)  0.96 (0.71-1.31)  1.00  1.14 (0.52-2.50)  0.94 (0.43-2.06)  0.87 (0.38-1.98)  1.00  1.08 (0.66-1.77)  1.12 (0.71-1.78)  1.02 (0.63-1.66)  1.00  0.96 (0.57-1.63)  0.85 (0.51-1.42)  0.87 (0.52-1.47) | 1.00  1.02 (0.74-1.40)  1.02 (0.76-1.40)  0.98 (0.72-1.34  1.00  1.19 (0.54-2.63)  1.02 (0.46-2.25)  0.94 (0.41-2.17)  1.00  1.15 (0.70-1.89)  1.21 (0.76-1.93)  1.11 (0.68-1.81)  1.00  0.98 (0.58-1.67)  0.87 (0.52-1.67)  0.90 (0.53-1.52) | 1.00  1.23 (0.87-1.74)  1.03 (0.74-1.45)  0.98 (0.69-1.41)  1.00  1.34 (0.56-3.20)  0.97 (0.39-2.23)  1.06 (0.42-2.69)  1.00  1.44 (0.84-2.45)  1.34 (0.81-2.24)  1.12 (0.65-1.95)  1.00  1.24 (0.70-2.22)  1.00 (0.56-1.77)  0.93 (0.51-1.69) | 1.00  1.08 (0.75-1.58)  0.87 (0.59-1.28)  0.88 (0.57-1.35)  0.62  1.00  1.46 (0.57-3.72)  1.21 (0.44-3.27)  1.89 (0.65-5.47)  0.11  1.00  1.30 (0.73-2.32)  1.17 (0.65-2.09)  1.04 (0.55-1.99)  0.88  1.00  1.31 (0.71-2.45)  1.05 (0.54-2.03)  1.06 (0.51-2.19)  0.39 |

*AHRR*, Aryl-hydrocarbon receptor repressor CI, confidence interval.

^a^ One individual are registered with up till nine causes of death, and might be included in analyzes with several causes of death.

*A priori* potential confounders were selected, and included in models 1) A crude model, 2) A model, adjusted for age at lung cancer diagnosis and sex. 3) ^b^A model, additionally adjusted for body mass index (kg/m2) ,ethnicity (European/others), TNM Classification of Malignant Tumors (TNM) (Stage I-IIII), histology of lung cancer (small cell lung cancer, adenocarcinoma, squamous-cell carcinoma, other non-small-cell lung carcinoma (NSCLC), performance status 4) ^c^A model additionally adjusted for smoking status (never/former/current smoker) and cumulative smoking (defined as 20 cigarettes/day per year, calculated from smoking intensity (number of cigarettes a day) and smoking duration (years)
