# Supplementary figures and images for: AHRR (cg05575921) methylation extent of leukocyte DNA and lung cancer survival
Source: PLoS One. 2019 Feb 7;14(2):e0211745. doi: 10.1371/journal.pone.0211745 (PMC6366765; doi:10.1371/journal.pone.0211745)

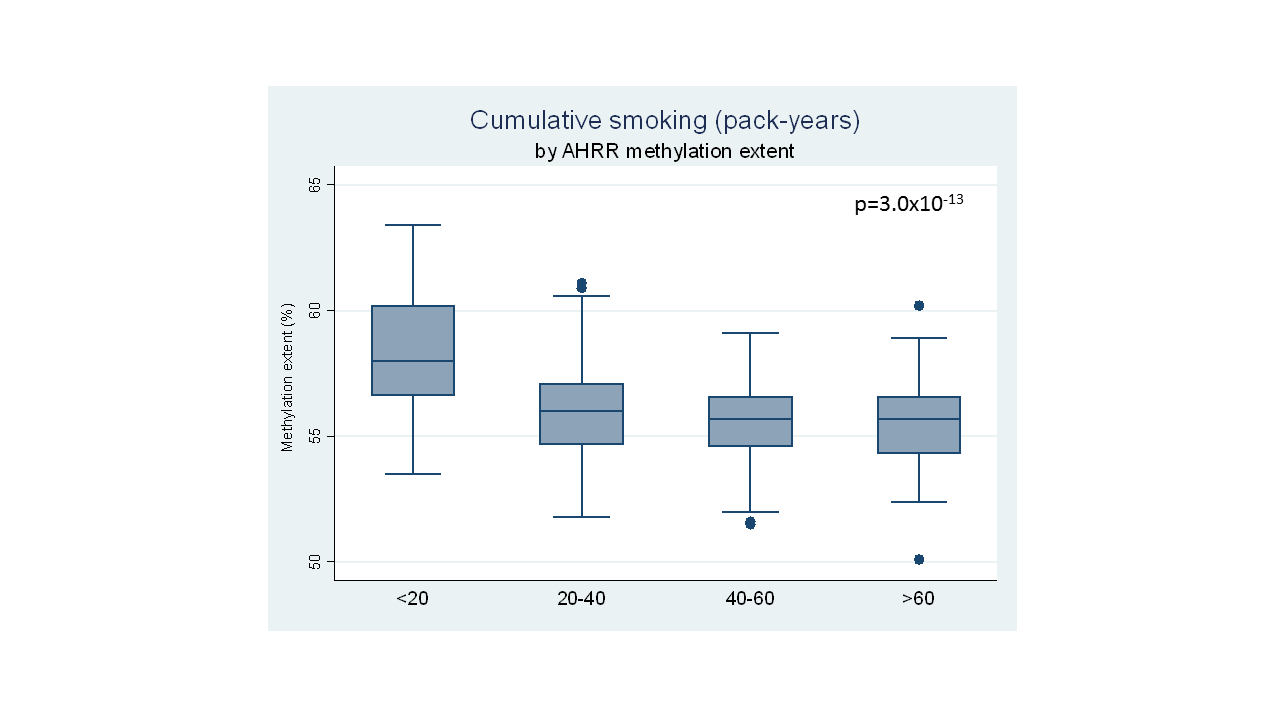

Supplement: S1 Fig — Values are median (p25 p75). AHRR, Aryl-hydrocarbon receptor repressor p-values (two-sided) were calculated with Pearsons X2-test for categorical values. (TIF) [file pone.0211745.s008.tif]

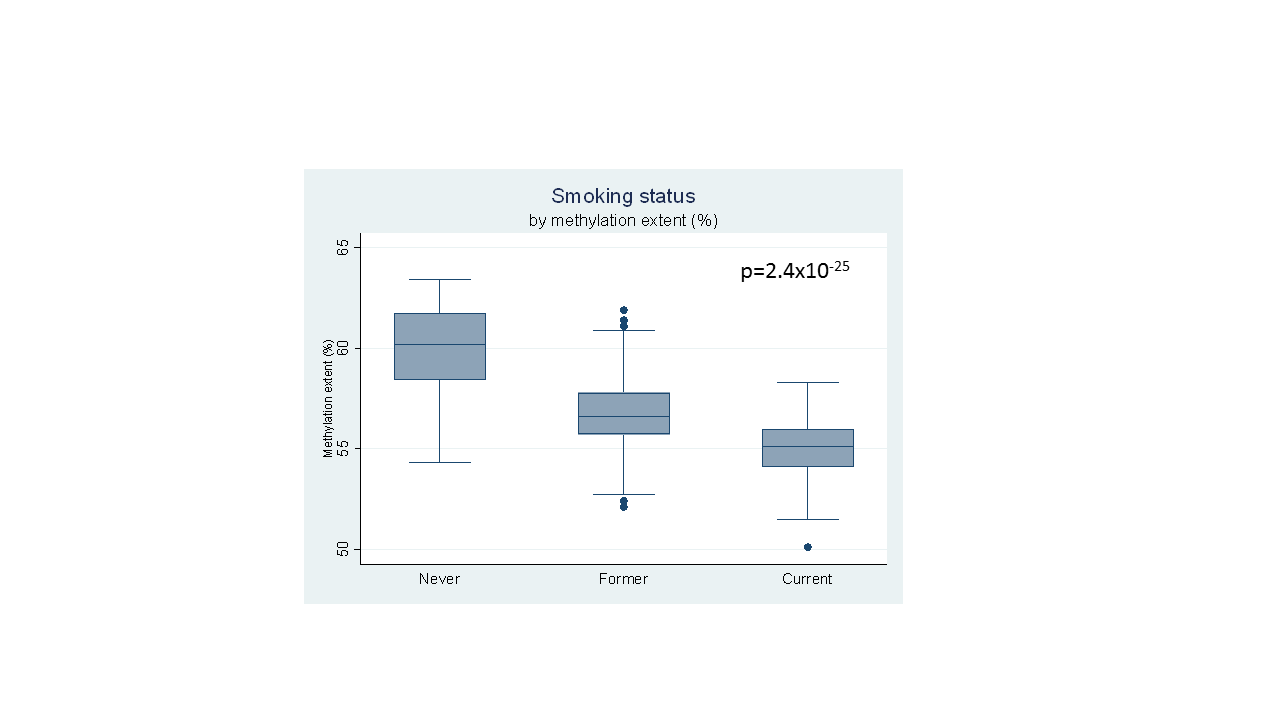

Supplement: S2 Fig — Values are median (p25 p75). AHRR, Aryl-hydrocarbon receptor repressor p-values (two-sided) were calculated with Pearsons X2-test for categorical values. (TIF) [file pone.0211745.s009.tif]

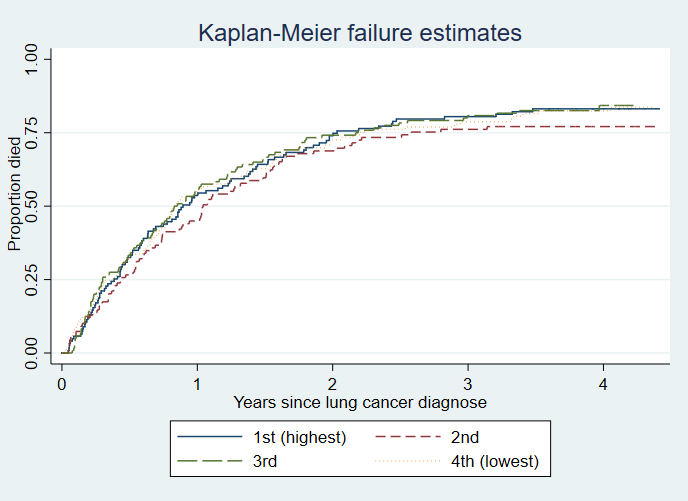

Supplement: S3 Fig — AHRR, Aryl-hydrocarbon receptor repressor 1st: Highest quantiles of AHRR (cg05575921) methylation extent. 4st: Lowest quantiles of AHRR (cg05575921) methylation extent. (TIF) [file pone.0211745.s010.tif]
